# Overexpressing 7-Hydroxymethyl Chlorophyll *a* Reductase Alleviates Non-Programmed Cell Death during Dark-Induced Senescence in Intact *Arabidopsis* Plants

**DOI:** 10.3390/biom11081143

**Published:** 2021-08-03

**Authors:** Xueyun Hu, Chu Zeng, Jinling Su, Imran Khan, Ahmad Zada, Ting Jia

**Affiliations:** 1 Joint International Research Laboratory of Agriculture and Agri-Product Safety of the Ministry of Education of China, Yangzhou University, Yangzhou 225009, China; xyhulab@yzu.edu.cn; 2 Key Laboratory of Plant Functional Genomics of the Ministry of Education, Yangzhou University, Yangzhou 225009, China; 3 College of Bioscience and Biotechnology, Yangzhou University, Yangzhou 225009, China; 172101127@yzu.edu.cn (C.Z.); 182102213@yzu.edu.cn (J.S.); dh18006@yzu.edu.cn (I.K.); dh19025@yzu.edu.cn (A.Z.)

**Keywords:** *Arabidopsis thaliana*, dark-induced leaf senescence, cell death, HCAR, chlorophyll degradation

## Abstract

Leaf senescence, the last stage of leaf development, is a well-regulated and complex process for investigation. For simplification, dark-induced leaf senescence has frequently been used to mimic the natural senescence of leaves because many typical senescence symptoms, such as chlorophyll (Chl) and protein degradation, also occur under darkness. In this study, we compared the phenotypes of leaf senescence that occurred when detached leaves or intact plants were incubated in darkness to induce senescence. We found that the symptoms of non-programmed cell death (non-PCD) with remaining green coloration occurred more heavily in the senescent leaves of whole plants than in the detached leaves. The pheophorbide *a* (Pheide *a*) content was also shown to be much higher in senescent leaves when whole plants were incubated in darkness by analyses of leaf Chl and its metabolic intermediates. In addition, more serious non-PCD occurred and more Pheide *a* accumulated in senescent leaves during dark incubation if the soil used for plant growth contained more water. Under similar conditions, the non-PCD phenotype was alleviated and the accumulation of Pheide *a* was reduced by overexpressing 7-hydroxymethyl Chl *a* (HMChl *a*) reductase (HCAR). Taken together, we conclude that a high soil water content induced non-PCD by decreasing HCAR activity when whole plants were incubated in darkness to induce senescence; thus, the investigation of the fundamental aspects of biochemistry and the regulation of leaf senescence are affected by using dark-induced leaf senescence.

## 1. Introduction

Leaf senescence, the final stage in leaf development, is important for plants to recycle and reallocate valuable resources to actively growing organs [1,2]. Leaf senescence is a complex but finely regulated process during leaf development [3]. During senescence, leaf cells undergo a dramatic transition, including the disorganization of chloroplasts and the degradation of chlorophyll (Chl) and photosystems, resulting in the loss of green color. Therefore, the breakdown of Chl is usually considered a biomarker of leaf senescence [4]. If Chl cannot be degraded when leaf senescence starts, the senescence process will be affected; thus, senescence processes will become disordered [4,5]. Free Chl and its metabolic molecules are deleterious molecules that generate reactive oxygen species because of their light-absorbing properties [6]. Previous studies have shown that light-dependent and light-independent non-programmed cell death (non-PCD)—shown by leaves dehydrated with photobleaching under light and with green color retained during dark-induced senescence—is induced by the accumulation of pheophorbide *a* (Pheide *a*) in Pheide *a* oxygenase (PaO) lacking *Arabidopsis* [5,7,8]. Pheide *a* was also shown to accumulate in a 7-hydroxymethyl Chl *a* (HMChl *a*) reductase (HCAR) knockout mutant with an unknown mechanism during dark-induced intact plant senescence, and non-PCD occurred [9].

During leaf senescence, Chl *b* needs to be converted to Chl *a*, and Chl *a* is subsequently degraded to colorless Chl catabolites inside chloroplasts. The major opinion about the Chl degradation pathway is that Chl *b* is firstly converted to HMChl *a* by two isozymes of Chl *b* reductase (CBR), non-yellow coloring 1 (NYC1) and NYC1-like (NOL) [10], and HMChl *a* is subsequently reduced to produce Chl *a* by HCAR [9]. The first step of Chl *a* degradation is catalyzed by magnesium (Mg)-dechelatase, encoded by Mendel’s green cotyledon gene, *STAY-GREEN* (*SGR*), which catalyzes Chl *a* to pheophytin *a* (Phetin *a*) [11]. The phytol chain of Phetin *a* is subsequently removed by Phetin Pheide hydrolase (PPH) and Pheide *a* is produced [12]. The porphyrin ring of Pheide *a* is oxygenolytically opened by PaO, producing red Chl catabolite (RCC) [5]. RCC is subsequently reduced by RCC reductase (RCCR) [13]. The resulting products, primary fluorescent Chl catabolite (pFCC) and hydroxyl-pFCC, the hydroxylation product of pFCC, are believed to be exported from chloroplasts and finally converted into nonfluorescent phyllobilins that are stored in the vacuole [14].

Developmental leaf senescence is a time-consuming biological processes, and many factors such as age, nutritional status, phytohormone content and other environmental factors affect the senescence progress of leaves. To obtain a synchronous process as a useful model for studying senescence, dark-induced leaf senescence has been frequently used to induce synchronous senescence, as many typical senescence symptoms such as Chl and protein degradation occur; however, there are also considerable differences in other key processes that take place [15]. For example, developmental leaf senescence is delayed in plants defective in the salicylic acid (SA) pathway, but dark-induced senescence is not obviously changed. Two methods are often used to study dark-induced leaf senescence. One method is keeping detached leaves in darkness with enough water to induce senescence, and the other is keeping whole plants grown in soil or medium in darkness to induce senescence [9,16]. To date, it is still unclear how the two dark-induced senescence methods may differ.

In this study, both detached leaves and intact plants were employed to induce leaf senescence in darkness. We found that Pheide *a* accumulated at obviously different levels, and varying severities of non-PCD occurred in leaves after treatment with these two methods. Compared to intact plants treated in darkness, darkness-treated detached leaves accumulated similar amounts of HMChl *a*, but much less Pheide *a*, and leaves showed lighter non-PCD symptoms. The HCAR knockout mutant showed a similar tendency, and overexpressing HCAR in wild-type (WT) plants obviously decreased the amount of HMChl *a* and Pheide *a*, and non-PCD was alleviated in the leaves of intact plants treated in darkness to induce senescence. We also found that during the dark treatment of intact plants, if the water content was higher in the soil, more HMChl *a* and Pheide *a* accumulated in the leaves. Taken together, we conclude that a high soil water content induces non-PCD through decreasing HCAR activity when intact plants are incubated in darkness to induce senescence, and a similar amount of HMChl *a* accumulation causes more Pheide *a* accumulation in the leaves of intact plants that are treated in darkness than in detached leaves that are treated in darkness to induce senescence.

## 2. Materials and Methods

### 2.1. Plant Material and Growth Conditions

The *Arabidopsis* T-DNA insertion mutants *hcar* (SALK_018790C), *nyc1* (SALK_091664) and *nol* (AL759262) were obtained from ABRC (Ohio State University) and GABI-Kat (Cologne, Germany). The *nyc1/nol* double mutant was generated previously [17]. Plants were grown in soil under long-day conditions (16 h light/8 h dark) with 80–100 μmol photons m^−2^ s^−1^ fluorescent light at approximately 23 °C. For plants grown in soil with approximately 70% moisture (70% of the water-holding capacity) or saturated moisture (100% of the water-holding capacity), relative water content of soil was controlled by giving water and measured weekly by using gravimetric analysis. Leaf numbers were counted from the bottom (oldest) to the top (youngest) of the plant.

### 2.2. Dark Treatment to Induce Senescence

Four-week-old *Arabidopsis* plants grown in soil were employed to induce senescence in darkness. Before dark treatment, the soil water content was adjusted to approximately 70% moisture or saturated moisture. Whole plants grown in soil were subsequently incubated in darkness for seven days. Then the leaves were harvested for further analysis. For detached leaves, dark-induced senescence was performed as follows: the No. 5–9 leaves were detached from 4-week-old *Arabidopsis* plants, subsequently placed on wet filter paper moistened with a buffer containing 3 mM MES (pH 5.8), and incubated in darkness for six days.

### 2.3. Plasmid Construction and Arabidopsis Transformation

Full-length complementary DNA (cDNA) of the *Arabidopsis* HCAR (At1g04620) gene was amplified, introduced into the Gateway entry vector pENTR4-Dual (Invitrogen, https://www.lifetechnologies.com/) and then introduced into the Gateway-compatible binary vector pEarleyGate100 by LR reaction [18]. The construct was transformed into *Agrobacterium tumefaciens* (strain GV3101) and subsequently transformed into *Arabidopsis* following the floral dip method [19]. HCAR-overexpressing transformants were screened by spraying the herbicide glufosinate ammonium (Basta).

### 2.4. Trypan Blue Staining

The trypan blue staining method was described previously [8]. Briefly, leaves were boiled for 1 min in lactophenol-trypan blue solution including 10 mL of lactic acid, 10 mL of glycerol, 10 g of phenol, 10 mg of trypan blue and 10 mL of distilled water and then decolorized overnight in chloral hydrate solution (2.5 g of chloral hydrate dissolved in 1 mL of distilled water).

### 2.5. Pigment Preparation and Chl Analysis

Chl and its metabolism intermediate molecules were extracted from the leaves before and after dark treatment by homogenization with precooled acetone [20]. The extracts were subsequently centrifuged for 5 min at 20,000× *g* at 4 °C, and the supernatant was analyzed by HPLC using a symmetric C8 column (150 mm in length, 4.6 mm in I.D.; Waters, Milford, MA, USA) according to the method of Zapata et al. [21]. Pigment concentrations were estimated from the absorption monitored at 410 nm. Standard Chl *a* and Chl *b* were purchased from Juntec Co. Ltd., Odawara, Japan, while Pheide *a* was purchased from Wako Pure Chemical Industries Ltd., Japan.

### 2.6. Immunoblot Analysis

Total protein was extracted from leaves using 10 volumes (*v*/*w*) of protein extraction buffer containing 50 mM Tris-HCl (pH 8.0), 12% (*w*/*v*) sucrose (Suc), 2% (*w*/*v*) lithium lauryl sulfate, and 1.5% (*w*/*v*) dithiothreitol. Before SDS-PAGE separation, all samples were mixed with an equal volume of 2× urea buffer containing 10 mM Tris-HCl (pH 8.0), 10% (*w*/*v*) Suc, 2% (*w*/*v*) SDS, 1 mM EDTA, 4 mM dithiothreitol, a small amount of bromophenol blue and 10 M urea and were electrophoresed on a 14% polyacrylamide gel and electroblotted to PVDF membranes. Samples were loaded based on the same weight of fresh leaves. As previously described, HCAR was detected using anti-HCAR antiserum raised in rabbits against recombinant *Arabidopsis* HCAR expressed in *E. coli* [9].

## 3. Results

### 3.1. More Pheide a Accumulated in WT Plants When Intact Plants Were Placed in Darkness to Induce Senescence

In order to compare senescence processes induced by dark incubation of detached leaves and whole plants, WT was firstly employed to investigate the phenotype. It was shown that the senescence process of leaves with similar age was slower in intact plants than in detached leaves. The observation is consistent with previous reports [16,22]. In addition, we noticed that non-uniform yellowing with a “green island” was visible in the senescent leaves of intact plants, while it was not obviously been observed in the senescent detached leaves. The “green island” phenotype implies that Chl degradation was affected during leaf senescence [23], which suggests us that Chl degradation derivatives should be analyzed. A previous report showed that both HMChl *a* and Pheide *a* accumulated in the senescent leaves of WT and *hcar* after the dark incubation of intact plants [9]. In order to investigate the correlation between the “green island” phenotype and the accumulation of HMChl *a* and Pheide *a*, *hcar* and *nyc1/nol* were also employed in this study, because *hcar* accumulates HMChl *a* and Pheide *a* during dark-induced senescence, and *nyc1/nol* is the mutant in which Chl *b* conversion to HMChl *a* is blocked and almost no HMChl *a* and Pheide *a* are accumulated.

**Figure 1:**
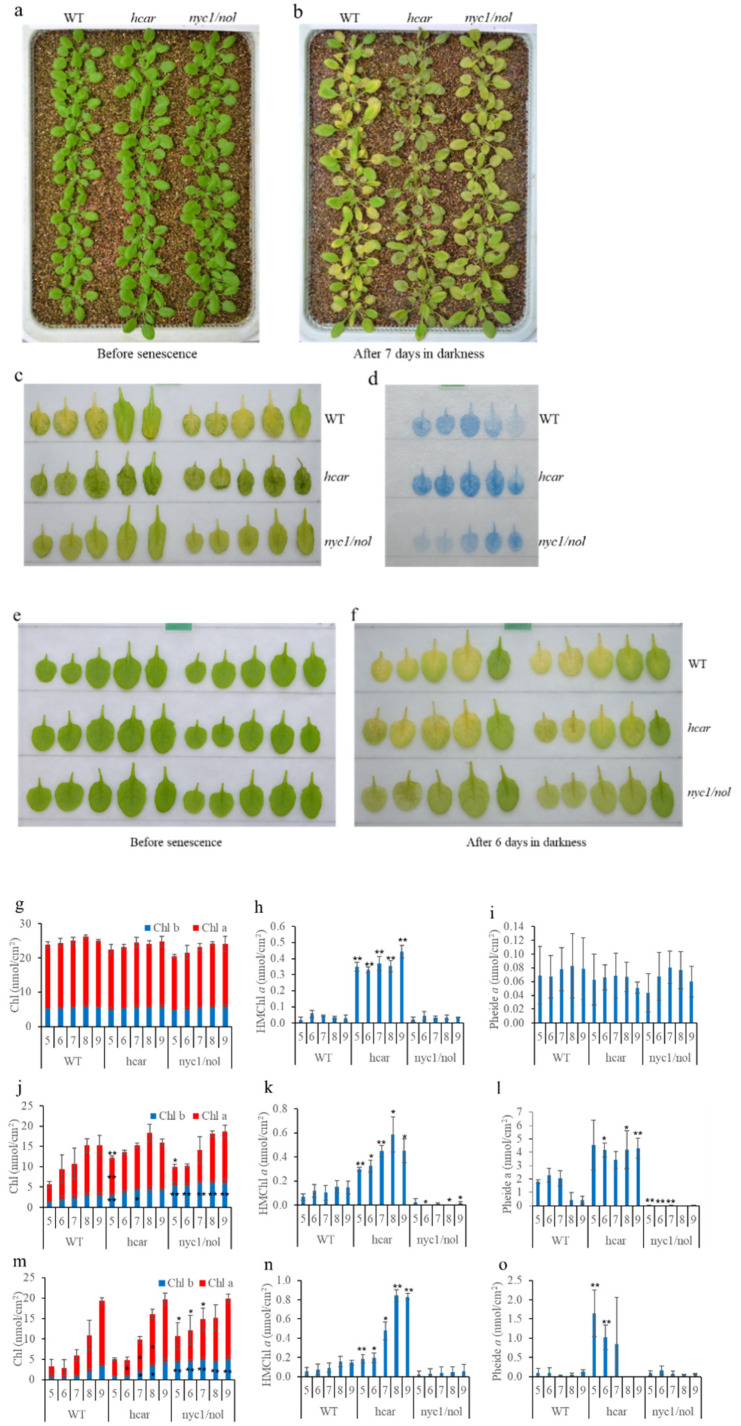
Phenotypic and Chl metabolic characterization of WT, *hcar* and *nyc1/nol* plants during dark-induced senescence. (**a**,**b**) Four-week-old WT, *hcar*, and *nyc1/nol* plants grown in soil before and after incubation in darkness for seven days. (**c**) No. 5–9 leaves (leaf numbers are counted from the bottom (oldest) to the top (youngest) of the plants) detached from dark incubated plants. (**d**) Detached No. 5–9 leaves from dark-induced plants stained with trypan blue. (**e**,**f**) Detached leaves from 4-week-old WT, *hcar*, and *nyc1/nol* plants before and after incubation in darkness for six days. (**g–i**), (**j–l**) and (**m–o**): Chl, HMChl *a*, and Pheide *a* content in No. 5–9 leaves (**g–i**) before, (**j–l**) after intact plant, and (**m**–**o**) after detached leaf incubation in darkness, respectively. Values are the means ± SD of three independent experiments. Asterisks indicate significant differences compared to the same number of WT leaves in the same condition (Student’s *t*-test, ** *p* < 0.01, and * *p* < 0.05). Asterisks within a blue-colored bar indicate a significant difference of Chl *b*, while asterisks within a red-colored bar indicate a significant difference of Chl *a*. Asterisks above bars indicate a significant difference of Chl, HMChl *a*, or Pheide *a*.

Four-week-old WT, *hcar* and *nyc1/nol* plants grown on soil were incubated in darkness for seven days (Figure 1a–c). Non-uniform yellowing with a “green island” was visible in the senescent leaves of WT plants, while *hcar* and *nyc1/nol* plants remained green with different appearances: *hcar* leaves shrank and dehydrated, while *nyc1/nol* leaves maintained a smooth surface without dehydration. After trypan blue staining, the results showed that some or all of the No. 5–7 leaves of WT plants were stained blue, indicating that cell death occurred heavily in the blue region (Figure 1d). The No. 5–9 *hcar* leaves were deeper blue than the WT leaves, while the No. 5–7 *nyc1/nol* leaves were lighter blue than the WT leaves. Detached leaves were subsequently kept in darkness for six days to induce senescence (Figure 1e,f). The WT leaves turned yellow almost without a “green island” and the *nyc1/nol* leaves turned pale green. However, the *hcar* leaves turned yellow with small regions of “green island”. The senescence phenotype of *nyc1/nol* was similar between whole plants and detached leaves that were treated in darkness to induce senescence; however, it was very different from the WT and *hcar* plants/leaves treated by the two methods.

Chl and its metabolic intermediate molecules in the leaves of WT, *hcar* and *nyc1/nol* plants were quantified. Before dark-induced senescence, the contents of Chl *a*, Chl *b*, and Pheide *a* were similar among WT, *hcar*, and *nyc1/nol* plants. HMChl *a* was significantly accumulated in *hcar* plants (Figure 1g–i). After keeping the intact plants in darkness for seven days, the Chl content in No. 5–9 leaves of WT, *hcar*, and *nyc1/nol* plants decreased (Figure 1j). The No. 6–9 leaves of all the plants retained a similar amount of Chl. More Chl was retained in the No. 5 leaves of *hcar* and *nyc1*/*nol* plants than that of WT plants, and the No. 5–9 leaves of *nyc1/nol* plants retained more Chl *b* than the No. 5–9 leaves of WT plants. The No. 5 and 7 leaves of *nyc1/nol* plants retained more Chl *b* than the No. 5 and 7 leaves of WT plants. Both HMChl *a* and Pheide *a* accumulated in WT and *hcar* plants, while they were almost lacking in *nyc1/nol* plants (Figure 1k,l). The amount of Pheide *a* in the No. 5–7 leaves of WT plants was almost half of that in the No. 5–7 leaves of *hcar* plants. Surprisingly, when detached leaves were incubated in darkness for six days, *hcar* leaves retained a similar amount of Chl to WT leaves, while No. 5–7 *nyc1/nol* leaves retained more Chl (Figure 1m). The amount of HMChl *a* in WT leaves was significantly lower than the amount of HMChl *a* in *hcar* leaves, and it was similar to the amount of HMChl *a* in WT leaves in whole plants treated in darkness (Figure 1k,n). A small amount of Pheide *a* was accumulated in WT detached leaves, and the amount of Pheide *a* in WT and *hcar* detached leaves was much less than that in the same number leaves of WT and *hcar* plants, respectively, when whole plants were treated in darkness to induce senescence (Figure 1l,o).

### 3.2. Non-PCD Was Not Visible in WT and Hcar Plants during Natural Leaf Senescence

**Figure 2:**
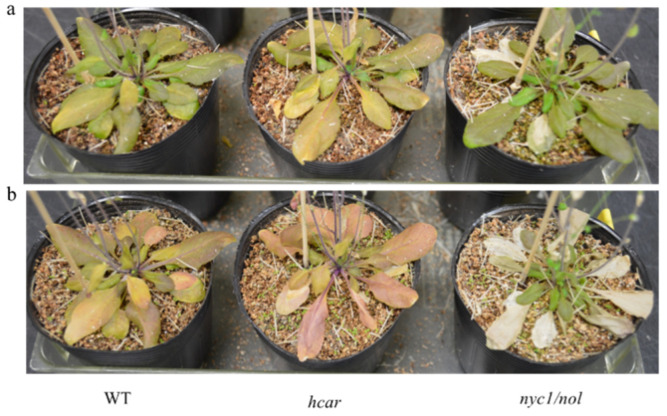
Phenotype of WT, *hcar*, and *nyc1/nol* plants during natural senescence: (**a**) 70-day-old WT, *hcar*, and *nyc1/nol* plants grown in soil; (**b**) 81-day-old WT, *hcar*, and *nyc1/nol* plants grown in soil.

Pheide *a* is a powerful photosensitizer, and its excessive accumulation in plants leads to the generation of reactive oxygen species (ROS) under light conditions, which ultimately causes cell death [8]. To analyze whether cell death occurs during plant senescence under light conditions, intact plants were grown under long-day conditions for more than 90 days, and the senescent phenotype was imaged at 70 and 81 days after sowing (Figure 2). The natural senescence phenotypes of WT and *hcar* plants were similar; the leaves turned yellow without visible cell death, while the leaves of *nyc1/nol* plants showed cell death with serious bleaching. These results imply that Pheide *a* can be degraded smoothly in the senescent leaves of WT and *hcar* plants. Otherwise, cell death should be observed in the senescent leaves of WT and *hcar* plants. For *nyc1/nol* plants, Chl *b* degradation is almost blocked during leaf senescence; therefore, photodamage occurs easily under illumination conditions.

### 3.3. A Great Water Content in Soil Causes More Severe Non-PCD Symptoms during Dark-Induced Whole Plant Senescence

**Figure 3:**
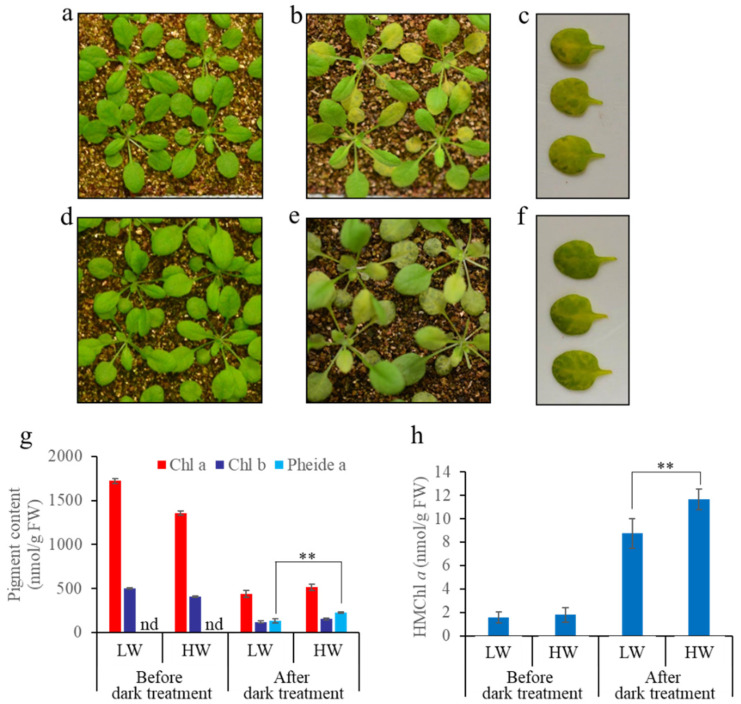
Phenotypic and Chl metabolic characterization of WT plants grown in soil with different water content during dark-induced senescence. (**a–c**) The phenotype of WT plants before (**a**) and after (**b**,**c**) 7 days of dark treatment. Plants were grown in soil with a low water content. (**c**) No. 5–6 leaves were detached from dark-treated WT plants. (**d–f**) The phenotype of WT plants before (**d**) and after (**e**,**f**) 7 days of dark treatment. Plants were grown in soil with a high water content. (**f**) No. 5–6 leaves were detached from dark-treated WT plants. (**g**) Chl and Pheide *a* contents in No. 5–6 leaves before and after intact plant incubation in darkness. (**h**) HMChl *a* content of leaves No. 5–6 before and after intact plant incubation in darkness. Values are the mean ± SD of three independent experiments. LW indicates soil with a low water content (approximately 70% moisture), and HW indicates soil with high water content (saturated moisture). Asterisks indicate significant differences (Student’s *t*-test, ** *p* < 0.01). nd indicates that the pigments content was not detected.

We tried to grow plants under different conditions and incubate the intact plants in darkness to induce senescence. We found that the water content of the soil for plant growth affected the senescence symptoms of WT plants during dark-induced senescence (Figure 3). After senescence, we found that a larger “green island” region appeared, and more Pheide *a* and HMChl *a* were detected in the plants grown in the soil with saturated moisture than in the soil with approximately 70% moisture (Figure 3g,h).

### 3.4. “Green Island” Symptoms Were Alleviated by Overexpressing HCAR in WT

**Figure 4:**
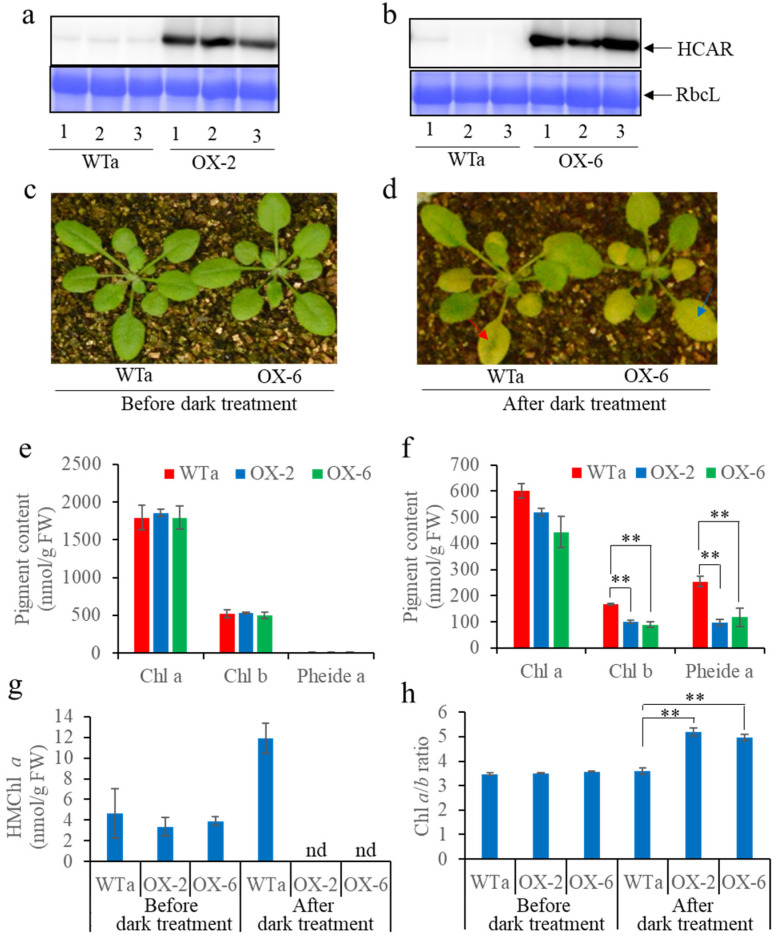
Phenotypic and Chl metabolic characterization of HCAR-OX and WT plants during dark-induced senescence. (**a**,**b**) HCAR protein abundance in WTa and HCAR-OX lines. Total leaf protein was separated by SDS-PAGE and subjected to immunoblot analysis with anti-AtHCAR antiserum. (**c**,**d**) Phenotype of HCAR-OX lines and WT plants before and after seven days of dark treatment. Red arrow indicates the “green island” symptoms of WT plants. The blue arrow indicates leaf senescence without “green island” symptoms of the HCAR-OX lines. (**e**,**f**) Chl and Pheide *a* content of No. 5–6 leaves before and after intact plant incubation in darkness. (**g**,**h**) HMChl *a* content and Chl *a*/*b* ratio of leaves No. 5–6 before and after intact plant incubation in darkness. Error bars indicate the SDs of three biological replicates. Asterisks indicate significant differences compared to BCG (Student’s *t*-test, ** *p* < 0.01). nd indicates the pigments content was not detected.

HMChl *a* and Pheide *a* accumulated in WT plants when whole plants were placed in darkness to induce senescence. To demonstrate whether the activity of HCAR was limited by the treatment, HCAR was overexpressed in WT plants. The Western blotting results showed that HCAR was overexpressed in the OX-2 and OX-6 lines (Figure 4a,b). There was no visible difference between the HCAR-overexpressing lines and WT plants during development (Figure 4c). After whole plants were treated in darkness for seven days, “green island” symptoms appeared in the senescent leaves of the WT plants, while the symptoms were alleviated by overexpressing HCAR (Figure 4c,d). Chl and metabolic intermediates were subsequently analyzed by HPLC. The results showed that, after seven days of dark-induced senescence, the HCAR-overexpressing lines retained less Chl *a* and *b*, had higher Chl *a/b* ratios, and accumulated less Pheide *a* than WT (Figure 4e,f,h). In WT plants, HMChl *a* was increased after senescence, while it was almost undetectable in the HCAR-overexpressing lines (Figure 4g). The results indicate that the activity of HCAR is insufficient in WT during dark-induced senescence, resulting in HMChl *a* accumulation and additive Pheide *a* accumulation. Therefore, non-PCD symptoms with “green islands” appeared during dark-induced senescence.

## 4. Discussion

Leaf senescence is a common phenomenon for plants. A loss of green color is one of the most noticeable properties of leaf senescence. Because of the complexity and time consumption of the natural senescence of leaves, dark-induced leaf senescence is often used as a model system to investigate leaf senescence. In this study, we found that incubating detached leaves in darkness can induce leaf senescence and overall yellowing, while incubating intact plants grown in soil in darkness caused the accumulation of Pheide *a,* and non-PCD occurred in the old leaves of WT plants. A higher content of water in the soil caused more severe non-PCD symptoms during whole plant senescence induced in darkness. Overexpressing HCAR in WT plants decreased the accumulation of HMChl *a* and Pheide *a*, and alleviated the non-PCD symptoms in the senescent leaves of plants incubated in darkness.

### 4.1. Non-PCD Symptoms Occurred in the Senescent Leaves of Intact Plants during Dark-Induced Senescence

As two often-employed methods to induce leaf senescence in darkness to investigate the mechanism of leaf senescence, incubating detached leaves or intact plants in darkness has not previously been investigated in detail. A previous study showed that the expression of senescence-associated genes (SAGs) was somehow different between detached leaves and whole plants treated in darkness to induce senescence [24]. As dark-induced leaf senescence occurs slowly in intact plants relative to detached leaves, molecular regulators (e.g., transcriptional regulators) have been suggested to be better visualized in such plants [16,22,25]. In this study, we found that by incubating whole plants in darkness for six days, non-PCD symptoms occurred heavily in the old leaves of the WT and *hcar* plants, while they were lighter in *nyc1*/*nol* plants (Figure 1d). Both WT and *hcar* plants accumulated HMChl *a* and Pheide *a*, and Chl metabolites caused the accumulation of Pheide *a* and non-PCD, respectively, during dark-induced leaf senescence [8,9]. These results imply that the non-PCD symptoms are caused mainly by Chl *b* degradation. Without Chl *b* degradation (in *nyc1/nol* plants), non-PCD symptoms were obviously alleviated (Figure 1d). By incubating detached leaves in darkness, we found that the senescent leaves of WT and *hcar* plants turned yellow, and much less Pheide *a* accumulated in the leaves than in the old leaves of whole plants treated in darkness (Figure 1). The senescent symptoms were similar to the natural senescence phenotype of WT and *hcar* plants, which did not show clear non-PCD symptoms and turned yellow at a similar rate (Figure 2). Non-PCD symptoms heavily affect the senescence processes of leaves; therefore, we conclude that incubating detached leaves in darkness could be a better method to induce leaf senescence than incubating intact plants in darkness for investigating programmed senescence of leaves.

### 4.2. HMChl a in Itself Is Insufficient to Induce the Accumulation of Pheide a and Cause Severe Non-PCD Symptoms during Dark-Induced Plant Senescence

It has been reported that HCAR-impaired mutants accumulate HMChl *a*, and they accumulate Pheide *a* after dark-induced senescence [9,26,27]. Two hypotheses have been provided to explain the reason why Pheide *a* degradation is affected during dark-induced senescence in HCAR-impaired mutants. One is that PaO is active only when HCAR is present. However, this possibility is unlikely, since *hcar* leaves turned to yellow without visible cell death, the phenotype of *pao* during natural senescence (Figure 2) [5]. Additionally, dark incubated *hcar* detached leaves turned to yellow with less Pheide *a* accumulation, compared to that in the leaves of dark incubated intact *hcar* plants (Figure 1). These results suggest that PaO activity is different in *hcar* plants under the two conditions. Another hypothesis is that PaO activity is inhibited by HMChl. This hypothesis is supported by the results that Pheide *a* did not accumulate in the *hcar-1*/*nyc1*/*nol* triple mutant. It would be difficult for HMChl to be a competitive inhibitor of PaO, because HMChl retains the phytol chain [9]. In an in vitro experiment, it was demonstrated that Pheide *b* inhibits PaO activity [28]. By catalyzing SGR and PPH, HMChl *a* can be converted to HMPhetin *a* and HMPheide *a* [23]. It is uncertain which intermediates inhibit the PaO activity. In this study, detached leaves and intact plants in darkness accumulated similar amounts of HMChl *a* in WT plants; however, the senescent leaves of whole plants in darkness accumulated much more Pheide *a* than detached leaves in darkness. A similar phenomenon happened in the *hcar* mutant as well. As a molecule causing leaf non-PCD [8], Pheide *a* accumulation caused more serious non-PCD symptoms in the senescent leaves of whole plants in darkness than in detached leaves in darkness (Figure 1). These results imply that the HMChl *a* level is not correlated with the amount of Pheide *a* or the severity of non-PCD symptoms. The accumulation of HMChl *a* is only the inducer of leaf non-PCD in darkness, because HMChl *a* accumulated in the leaves of *hcar* plants; however, before dark treatment, there was no visible difference between WT and *hcar* plants (Figure 1) [9]. No obvious non-PCD symptoms occurred in natural senescent leaves of *hcar* plants (Figure 2). Unidentified environmental or developmental changes might induce the accumulation of Pheide *a* and the occurrence of non-PCD in darkness.

### 4.3. Soil Water Content Affects Chl Degradation by Affecting HCAR Activity

In this study, we found that plants grown in soil with a higher content of water—saturated moisture compared to 70% moisture—accumulated more HMChl *a* in their senescent leaves, accompanied by greater Pheide *a* accumulation, and more serious non-PCD symptoms occurred if whole plants were employed for dark-induced senescence (Figure 4). These results imply that the activity for HMChl *a* degradation is limited during dark-induced Chl degradation under these conditions, and the limitation is more serious if whole plants are grown in soil with a higher water content. Soil or air humidity may be an important environmental factor that affects HMChl *a* degradation during dark-induced whole plant senescence. Since Chl degradation is slower and more HMChl *a* accumulates with a higher soil water content during dark-induced intact plant senescence, HCAR activity is lower when the soil water content is higher. However, the mechanism needs further investigation.

### 4.4. HCAR Is Not Essential for Chl b Degradation

To date, HCAR is the sole enzyme that has been discovered to catalyze HMChl *a* to produce Chl *a* [9]. Previous research showed that staying green and non-PCD symptoms occurred in the senescent leaves of *hcar* mutants when whole plants were in darkness [9]. It is also true in our experiments, however, that the leaves of the *HCAR* knock-out mutant also turned yellow when detached leaves were incubated in darkness to induce senescence (Figure 1e,f), although they stayed slightly greener than that of the WT. In addition, the *HCAR* knock-out mutant was grown in soil for natural senescence, and the leaves also turned yellow (Figure 2). These results indicate that HCAR is an unessential enzyme for Chl *b* degradation in *Arabidopsis*. Whether another enzyme possesses the activity of catalyzing the conversion from HMChl *a* to Chl *a* needs to be addressed in the future. Another possibility is that without HCAR, HMChl *a* can be converted to produce HMPhetin *a* and HMPheide *a* by the catalyzing of SGR and PPH [23]. PaO may catalyze HMPheide *a* to further degradation. 

## Data Availability

The data presented in this study are available on request from the corresponding author.
